# Skin invasion and prognosis in node negative breast cancer: a retrospective study

**DOI:** 10.1186/1477-7819-6-10

**Published:** 2008-01-30

**Authors:** Keiichiro Tada, Hidetomo Morizono, Kotaro Iijima, Yumi Miyagi, Seiichiro Nishimura, Masujiro Makita, Rie Horii, Futoshi Akiyama, Takuji Iwase

**Affiliations:** 1 Department of Breast Surgery, Cancer Institute Hospital, Tokyo, Japan; 2 Department of Pathology, The Cancer Institute of the Japanese Foundation for Cancer Research, Tokyo, Japan

## Abstract

**Background:**

The impact of skin invasion in node negative breast cancer is uncertain.

**Methods:**

We determined the prognosis in 97 node negative breast cancer patients (case group) who had tumors with skin invasion. Then we compared these patients with 4500 node negative invasive breast cancer patients treated surgically in the same period.

**Results:**

Patients with skin invasion tended to be older, had more invasive lobular carcinoma and larger tumor size, and were less likely to have breast conserving surgery than those in the control group. The 5-year disease-free survival rate in the case group was 94.0%. There was no significant difference in the 10-year disease-specific overall survival rates in terms of skin invasion in node negative patients (90.7% in the case group, 92.9% in the control group; p = 0.2032).

**Conclusion:**

Results suggest that skin invasion has no impact on survival in node negative invasive breast cancer patients. The adjuvant regimens which the individual institute applies for node negative breast cancer should be used regardless of skin invasion.

## Background

It is well known that the number of metastatic lymph nodes is closely associated with the prognosis of breast cancer patients[1,2]. However, some node-negative breast cancer patients, who are believed to have good prognosis, experience recurrent disease. Therefore, it is important to know the prognostic factors in node-negative breast cancer patients.

Skin invasion is one of the classical pathological factors that is associated with prognosis [3]. The T4b category, according to the TNM classification, includes tumors with edema, ulcers, and satellite skin nodules that are signs related to skin invasion of the cancerous lesions[4]. In usual clinical practice, we sometimes encounter histopathological skin invasion in node negative breast cancer patients. However there are few reports concerning this issue. In this article, we investigate the significance of skin invasion as a prognostic factor in node negative breast cancer patients.

## Patients and methods

From 1983 to 1999, 8013 patients who had surgical treatment for breast cancer were registered in our institute database. Among these cases, we looked for breast cancer patients fulfilling the following requirements: skin invasion determined histopathologically, node negative disease, no distant metastasis, no primary chemotherapy and curative treatment. Cases of synchronous bilateral breast cancer were excluded from this study. There were 97 patients who met all these criteria. Then, we studied these patients in terms of demography, clinical and pathological tumor characteristics, and prognosis.

Because this study is retrospective, accurate information on survival status, especially survival with recurrence, is difficult to obtain. However, we could obtain long-term results of survival or death. Furthermore, we could obtain the etiology of death. Therefore, we plotted the survival curve based on disease-specific overall survival using the Kaplan-Meier method. Only death caused by breast cancer was considered. Those who died from other causes, as well as the survival cases, were considered censored cases.

As a control group, we used all node negative invasive breast cancer patients treated surgically during the same period. A total of 4567 cases were found in our database. The number of resected lymph nodes is closely associated with the accuracy of the determination of node-negativity [5]. Therefore, 67 patients with five or fewer resected lymph nodes were excluded from the study. As a result, we analyzed the data for 4500 patients for the control group. We studied these patients in terms of demography, clinical and pathological tumor characteristics, and prognosis. The overall survival curve of this group was plotted as described above. In order to compare baseline characteristics and treatment types between the case and control groups, Student's t test was used for age, tumor size, and the number of resected lymph nodes. A chi square test was also used for comparison of other factors. The comparison of groups in terms of survival was made with the log rank test. Statistical significance of a two-sided test was defined as a p-value less then 0.05. The SPSS 11.0 software package was used for these calculations.

## Results

The baseline characteristics of 97 cases with node negative skin invasion are shown in Table 1. The 97 patients included 96 women and one man. The mean age was 58.8 (range: 30–86). The mean size of tumor was 3.0 cm (range: 0.8–13.0 cm). Although the histological subtypes varied significantly, cases of invasive ductal carcinoma (77 cases, 79.4%) were prominent. Forty-nine cases (50.5%) had estrogen receptor positive disease, 30 cases (30.9%) had estrogen receptor negative disease, and 18 cases (18.6%) were in the receptor unknown group. Clinical evaluation of the skin overlying the tumor is summarized in Table 2. Most cases had signs in the overlying skin. However, 4 cases (4.1%) could not be evaluated for skin involvement preoperatively. The type of surgical treatment and post-operative treatment is summarized in Table 3. Because these cases occurred several years ago, 90 cases (92.8%) had total mastectomy. All cases had axillary resection, and the median number of removed lymph nodes was 22 (Range; 5 to 70). Twenty-eight cases had chemotherapy, such as cyclophosphamide, methotrexate, and fluorouracil, or the oral derivatives of fluorouracil. Forty patients (41.2%) had endocrine therapy. All of them were given tamoxifen. Radiation therapy was given to 3 (3.1%) patients.

**Table 1:** Demography and tumor characteristics

		Case		Control		
Gender	Female	96	(99.0%)	4485	(99.7%)	p = 0.290
	Male	1	(1.0%)	15	(0.3%)	
Age	Mean	58.8		52.8		p < 0.001
	Range	30–86		23–91		
Tumor size (cm)	Median	3		2.2		p < 0.001
	Range	0.8–13.0		0–20		
Nodal status	Node Negative	97	(100%)	4500	(100%)	
	Node Positive	0	(0%)	0	(0%)	
Histological subtype	Invasive ductal	77	(79.4%)	3941	(87.6%)	p = 0.002
	Invasive lobular	12	(12.4%)	194	(4.3%)	
	Mucinous	5	(5.2%)	216	(4.8%)	
	Other	3	(3.1%)	145	(3.3%)	
Estrogen receptor	Positive	49	(50.5%)	1608	(35.7%)	p < 0.001
Status	Negative	30	(30.9%)	1132	(25.2%)	
	Unknown	18	(18.6%)	1760	(39.1%)	

**Table 2:** Clinical findings in skin in the ipsilateral breast

Dimpling	34	(35.1%)
Tumor fixing to the skin	30	(30.9%)
Nipple retraction	15	(15.5%)
Ulcer	6	(6.2%)
Edema	1	(1.0%)
Post biopsy	3	(3.1%)
No abnormal findings	4	(4.1%)

**Table 3:** Treatment

		Case		Control		
Operation	Total mastectomy	90	(92.8%)	3504	(77.9%)	p < 0.001
	Partial mastectomy	7	(7.2%)	963	(21.4%)	
	Unknown	0	(0%)	33	(0.7%)	

Number of removed	Median	22		22		p = 0.630
lymph nodes	Range	5–70		5–125		

Chemotherapy	Yes	28	(30.0%)	1051	(23.4%)	p = 0.127
	No	69	(70.0%)	3449	(76.6%)	

Endocrine	Yes	40	(41.2%)	1591	(35.4%)	p = 0.138
therapy	No	57	(58.8%)	2909	(64.6%)	

Radiation therapy	Yes	3	(3.1%)	359	(8.0%)	p = 0.081
	No	93	(96.9%)	4141	(92.0%)	

For the control group, the baseline characteristics and treatment types are listed in Tables 1 and 3, respectively. Gender, the number of resected lymph nodes, chemotherapy, and endocrine therapy were not significantly different between groups. On the other hand, older patients, larger tumor size, more histological subtypes of invasive lobular carcinoma, more unknown receptor status, and more partial mastectomies were observed for the case group with statistical significance.

**Figure 1:**
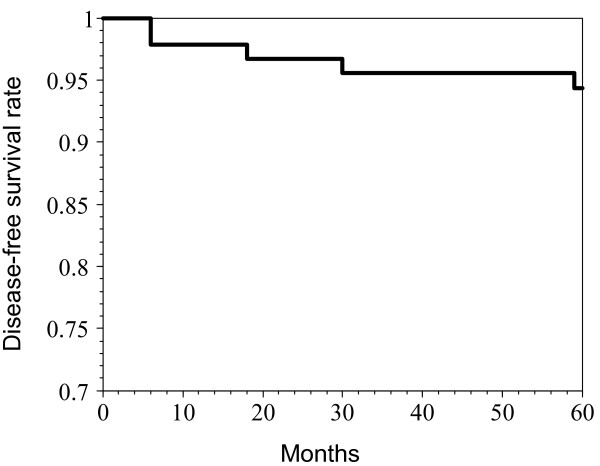
The disease-free survival curve for 97 skin invasive node negative breast cancer patients.

**Figure 2:**
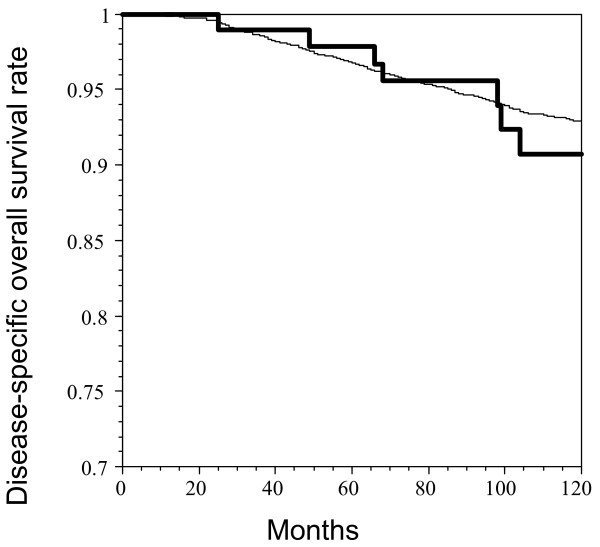
The disease-specific overall survival curve for 97 skin invasive node negative patients (bold line) compared with that for 4500 node negative patients (fine line).

The disease-free survival curve of these 97 patients is shown in Figure 1. The 5-year disease-free survival rate was 94.0%, with a median follow up of 90 months. The 90-month disease-free survival rate was 84.0%. The disease-specific overall survival of these 97 patients is compared with the control group in Figure 2. There was no significant difference between these 2 groups. The 10-year overall survival rates were 90.7% in the case group and 92.9% in the control group (p = 0.2032). The median follow up times were 118 months in the case group and 116 months in the control group.

## Discussion

This study demonstrates that skin-involving node negative breast cancer patients had a 5-year disease-free survival rate of 94.0% and a 10-year disease-specific overall survival rate of 90.7%. The latter figure was comparable with that of overall node negative breast cancer patients (92.9%, p = 0.2032). These results suggest that skin invasion has no effect on survival in node negative breast cancer patients.

There were some differences in tumor characteristics and treatment type between the case and the control groups. The mean tumor size in the case group was larger than that in the control group. This fact means that there was less frequent breast-conserving surgery, and more frequent unknown receptor status in the case group. Previously, the receptor status was determined based on enzyme immunoassay (EIA). Because EIA requires a fresh sample of tumor, receptor status tends to be unknown when the cancerous lesion is too small.

Invasive lobular carcinoma was more common in the case group. The patients with this subtype of breast cancer tended to be older, have a larger tumor size, and have a lower rate of lymph node involvement [6]. These characteristics might contribute to the differences between the case and the control groups in our study. Although all these differences have to be taken into consideration, we believe that these disparities do not affect the survival analysis significantly.

The nodal status is the most reliable prognostic factor, and a negative node finding is associated with the most favorable prognosis. However, distant metastasis can develop even in these node negative patient groups. Therefore, we have to seek other reliable prognostic factors independent of nodal status.

The significance of skin invasion in node negative breast cancer is uncertain. Tumors with signs of skin invasion, such as edema, ulcers, and satellite nodules, are classified as T4 category in TNM classification. Patients with a T4b tumor are considered as having advanced disease. Furthermore, Perrone *et al*. reported that skin invasion was one of the prognostic factors in breast cancer[3]. On the other hand, it has been reported that skin invasion loses prognostic significance in multivariable analysis, and only nipple invasion has impact on prognosis[7]. Our data suggest that skin invasion is not a prognostic factor independent of nodal status.

We accept that our study has limitations. It is a retrospective study, has a small number of cases, and does not include strictly T4 tumors. However, we believe that our findings can guide clinical practice in breast cancer.

Many prognostic factors in breast cancer have been studied recently. The St. Gallen consensus advocates prognostic factors other than nodal status, such as vascular involvement, receptor status, nuclear grading, HER2 status, and age and size of tumor [8]. Furthermore, recent advances in molecular biology have led to identification of biological markers that are associated with biological activities of the tumors. Gene-expression-profiling studies [9] including urokinase-type plasminogen activator: plasminogen activator inhibitor type-1 complex [10], estrogen receptor, progesterone receptor [11], cyclin E [12], and HER2 [13] are the results of these advances. However, we believe that classical histopathological evaluation is still important because of its ubiquitous use and good cost-benefit balance.

Sometimes skin invasion cannot be predicted preoperatively. Based on our findings, dimpling alone can be a clue for skin invasion. Skin invasion is important for management of the overlying skin in the surgical treatment. Whether in total mastectomy or in breast conserving treatment, complete resection is essential for avoiding the risk of local recurrence [14]. Removal of overlying skin is necessary when skin invasion is predicted.

## Conclusion

Our study suggests that skin invasion has no impact on survival in node negative invasive breast cancer patients. The adjuvant regimen which the individual institute determines for node negative breast cancer should be applied to skin invasive node negative breast cancer patients.

## Competing interests

The author(s) declare that they have no competing interests.

## Authors' contributions

**KT **designed the study, searched the literature, and drafted the manuscript. **FA **and **RH **contributed to the pathology analysis and pathological part of the manuscript drafting. **HM, KI, YM, SN, MM**, and **TI **participated in this study's design and coordination, and helped to collect data. All authors read and approved final manuscript.
